# Chemical physics: The standing of a mature discipline

**DOI:** 10.1186/1752-153X-1-6

**Published:** 2007-03-02

**Authors:** Eduardo A Castro

**Affiliations:** 1 INIFTA, Theoretical Chemistry Division, Sucursal 4, C.C. 16, La Plata 1900, Buenos Aires, Argentina

## Abstract

It is always promising and enticing to start a new editorial task in the scientific arena and the launch of the Chemistry Central Journal is no exception. The different thematic sections making up this journal are quite representative of the whole chemistry enterprise. However, one of them has a special relevance. In fact, Chemical Physics (CP) is the most general and it embodies a wide diversity of issues. Of particular importance at the launch of this groundbreaking new journal is the confidence of the Section Editor in BioMed Central (owners of Chemistry Central) as publishers, and from Chemistry Central to its Editorial Board. I feel deeply grateful for this new assignment and I hope to be able to perform a thorough job in editing this section. Below, I make my request to you as potential authors and reviewers.

## Background

It is always promising and enticing to start a new editorial task in the scientific arena and the launch of the *Chemistry Central Journal *is no exception. The different thematic sections making up this journal are quite representative of the whole chemistry enterprise. However, one of them has a special relevance. In fact, Chemical Physics (CP) is the most general and it embodies a wide diversity of issues. Of particular importance at the launch of this groundbreaking new journal is the confidence of the Section Editor in BioMed Central (owners of Chemistry Central) as publishers, and from Chemistry Central to its Editorial Board. I feel deeply grateful for this new assignment and I hope to be able to perform a thorough job in editing this section. Below, I make my request to you as potential authors and reviewers.

Perhaps it is tempting to try to give an embracing definition of CP. However, there is no well-defined set of subject matter that constitutes CP, and any attempt to give a description of the subject in terms comparable with those used to define Organic or Inorganic Chemistry would only be misleading. In fact, CP is not a branch of chemistry that stands beside Organic and Inorganic Chemistry. It is rather to be thought of as an approach which is applicable to all branches of Chemistry. A quite general introductory definition is that CP deals with those chemical phenomena which can be studied quantitatively. It is based on the accurate data of experiment, and is thus essentially a laboratory subject. Data, however, when systematically examined, suggest hypotheses; hypotheses cohere into theories; theories, in turn, gain in power and clarity when they can be expressed in mathematical form. Each theory which thus emerges must be critically tested in the light of the facts it was intended to interpret, where possible, in the light of such new experiments as may be suggested by it. This is the order of development which has scientific sanction and is the one which is particularly stressed.

In practice, the principal activities that are classified as chemical physical are usually easily recognized. These activities are generally concerned with the phenomena that arise from, and are, hopefully, interpretable in terms of, molecular behavior. The chemical physicist tends to apply a quantitative treatment to the systems with which he/she deals. The attitude with which the subject is approached is that CP is primarily the study of the molecular world and the explanation of chemical phenomena on the basis of molecular behavior. It is this attitude that is found in the laboratories of the world where research and development in CP are being actively pursued and in the current courses where this discipline is taught.

These comments make clear why CP is important as a discipline and why it currently interfaces with all chemistry areas. The research topics in the CP field include traditional disciplines such as spectroscopy, kinetics, statistical mechanics, thermodynamics, molecular structure, electrochemistry and macromolecules, among others. But it is also well deserving to mention other relatively new branches such as materials, surface phenomena, irreversible processes, chaos, quantitative structure-property (or activity) relationships, medicinal chemistry, theoretical chemistry, green chemistry, among some of the most relevant ones.

Now I try to illustrate this view of CP research by briefly presenting some illustrative examples of actual significant issues of topical research areas. Naturally, it is not possible to describe here all of them, but I deem that hopefully the reader can grasp the wide variety of approaches and the diversity of chemical phenomena which are being currently studied in the CP field.

## Photonic Crystals

Photonic crystals are the electromagnetic analog of semiconductor crystals. They are artificial crystal structures that do for electromagnetic waves what semiconductors do for electron waves. In today's world, electronic semiconductors are the basis for the micro-electronic, telecommunications, and computer industries. We are just now beginning to understand the exciting potential of their electromagnetic cousins for tomorrow's world. The powerful analogy between photonic and semiconductor crystals has unleashed the collective scientific imagination of many creative chemical physicists, engendering a profusion of synthetic electromagnetic crystal structures. These usually have an electromagnetic bandgap, a band of frequencies in which electromagnetic waves are forbidden. Various 2-dimensional and 3-dimensional photonic crystals structures have now been conceived for application in high capacity optical fibers, color pigments, and specially nanophotonic integrated circuits that might be included in standard microchips. It appears likely that circuit concepts derived from these sort of crystals can be extended right back to optical frequencies, where they emerge as so-called "plasmons", the optival frequency currents that can flow on metallic surfaces. Such ultra-miniature LC circuit arrays, smaller than an optical wavelength, may eventually represent the ultimate end point of photonic crystal miniaturization [1,2].

## Single-Molecule Conductivity

The goal of building sophisticated electronic devices from individual molecules has spurred studies of single-molecule rectification [3], nanotube-based transistors [4], and negative differential resistance from small collections of molecules [5]. The primary problems facing the molecular electronic designer are measuring and predicting electron transport. Molecular electronics will also require reliable molecular wires to carry signals from one molecular circuit element to another. A key requirement in all these studies is the ability to measure the conductivity of a single molecule. To do so, one must connect a macroscopic current source and volt meter to each end of a single molecule. Molecular electronics is thus much about contacts. Ideally, these contacts should be ohmic so that any nonlinearity in the conductivity of the wire can be correctly attributed and studied. They must also be low in resistance to ensure that the properties measured are those of the molecule and not those of the molecule-contact interface. Moreover, the medium surrounding and supporting the molecule must be several orders of magnitude more insulating than the molecule itself because the contact area of the support with the electrical contacts is often much greater than that between the electrical contacts and the molecule.

Cui et al. presented a simple meted for making good electrical contacts to variable length organic molecules [6]. They used thiol groups to make well-defined chemical bonds to a gold base electrode and a gold nanoparticle top electrode. Contact to the nanoparticle was made through physical contact with a gold-coated atomic force microscopy tip. The molecule is thus covalently bonded to gold at both ends. Isolation of the current to a single molecule was produced by diluting the dithiol functionalized alkanes. Hence, even though the nanoparticle is much larger than the molecule of interest, the number of actual contacts per particle is small and peaks at a single contact per particle.

## Accurate Theoretical Thermochemistry

The first-principles evaluation of the binding energies of molecules to chemical accuracy (i.e. ± kcal/mol) is one of the most challenging problems in computational quantum chemistry. Dramatic progress has been made in this regards in the last two decades and the rigorous demands placed on the theoretical methods to achieve this goal are now well understood. In principle it is now known how to compute the binding energies and other thermochemical properties of most molecules to very high accuracy. This can be achieved by using very high levels of correlation, such as that obtained with coupled cluster or quadratic configuration interaction methods, and very large basis sets containing high angular momentum functions. The results of these calculations are then extrapolated to the complete basis set limit and corrected for some smaller effects such as core-valence and relativistic effects. Unfortunately, this approach is limited to small molecules because of the ~N^7 ^scaling (with respect to the number of basis functions N) of the correlation methods and the need for very large basis sets. An alternative approach applicable for larger molecules is to use a series of high-level correlation calculations, such as QCISD(T), MP4 or CCSD(T), with moderate sized basis sets to approximate the result of a more expensive calculation. The Gaussian-n series exploits this idea to predict thermochemical data. In addition, molecule-independent empirical parameters are used in these methods to estimate the remaining deficiencies in the calculations. Such an approach using high-level corrections (additive parameters that depend on the number of paired and unpaired electrons in the system) has been quite successful, and the latest version, Gaussian-3 (G3) theory, achieves an overall accuracy of 1 kcal/mol for the G2/97 test set. Petersson *et al*. Have developed a related series of methods, referred to as complete basis set (CBS) procedures, for the evaluation of accurate energies of molecular systems. The central idea in the CBS methods is an extrapolation procedure to determine the projected second-order (MP2) energy in the limit of a complete basis set. Several empirical corrections, similar in spirit to the higher-level correction used in the Gaussian-n series, are added to the resulting energies in the CBS methods to remove systematic errors in the calculations. Another approach to calculation of thermochemical data that has been proposed is scaling the calculated correlation energy using multiplicative parameters determined by fitting to experimental data. Finally, hybrid density functionals are being used increasingly to predict the thermochemistry of molecules with reasonable accuracy [7]

## Supramolecular Chemistry

Sometimes even molecules need to dress for success. In fact, recently chemists at the University of California, Los Angeles, and London's Imperial College have come up wit a new classs of mechanically interlocked structures called suitanes. As the name suggests, the compounds feature one molecule dressed in another, with no covalent link between the two [8]. A suitane's basic components consists of a single-molecule "body" with two or more rigid limbs and a close-fitting, all-in-one suit molecule that encompasses the body's torso. The body molecule's limbs protrude outward from the suit, just as arms and legs stick out of real-world clothes. Shorts are a good human-scale analogy for a suit[3]ane because three so-called limbs -two legs and a torso- poke out from the clothing; a T-shirt is analogous to a suit[4]ane; and the one-piece infant romper known as a onesie is akin to a suit[5]ane. The synthesis of this new class of molecules was guided by previous computational chemistry studies and the group of researchers based the body of their suit[2]ane on a slim phenyl waist flanked by two bulky anthracene units on either side. On the other side of each anthracene moiety, they placed a secondary ammonium salt intended to hydrogen bond with pyridine units in two complex crown ether structures that make up most of the suit. Now what remain to be done is a sort of inverse theoretical study about the nature of non-existing chemical bond among the different parts of this supramolecular species, although they are in close contact each other.

## Solid State Chemistry

Small molecules can be trapped, like hostages in a prison cell, by networks of cages formed from another compound. The resulting solids, known as clathrates, or inclusion compounds, have been known about for more than 150 years, but have recently gained media attention for their potential to trap the greenhouse gas carbon dioxide [9]. Many types of clathrate have been identified, and it seemed that little remained to be discovered. But recently, Karau and Schnick describe the synthesis of a clathrate with a unique cage structure [10].

Clathrates are formed when a host compound encloses guest molecules without using strong ionic interactions or covalent bonds. The name "clathrate" was also applied to inclusion compounds with lattices constructed from covalent bonds. As exemplified by a series of silica structures that are known as clathrasils. Nanoporous frameworks of silicon or germanium semiconductors and even some networks of inorganic covalent salt complexes have also been described as clathrates. The burgeoning interest in this sort of materials is fuelled by their potential uses in catalysis, gas storage and separative membranes.

In true clathrates, a tetrahedral framework of hydrogen bonds creates voids in which guest species are trapped. A good example of this is methane hydrate, discovered deep on the oceans, in which methane molecules are embedded in a lattice of water. The resulting material looks like dirty ice, and could be a source of fossil fuel. Only ecxtremely weak interactions are required for clathrates to hold molecules -for example, the gases xenon and krypton are renowned for being generally unreactive, but both can act as guests in inclusion compounds. The voids are often in the shape of symmetric polyhedra, such as a pentagonal dodecahedron.

Magnetorheological (MR) materials comprises magnetizable particles dispersed in a nonmagnetic host. In MR fluids, the particles are usually micrometer-sized carbonyl iron powers in a host medium such as natural or synthetic oil and in magnetic powders, the fluid medium is air. These materials transform from freely flowing to weakly solid under an applied magnetic field, a dramatic phenomenon that is the basis for a variety of commercial applications of these controllable or "smart" materials. In MR elastomers, the particles are locked into viscoelastic solids, but these composites exhibit substantial magnetostriction. While MR fluids are similar to ferrofluids, nanometer-sized colloidal dispersions of single-domain magnetic particles, the two materials possess rather different behaviors. In fact, ferrofluids display only small increases in viscosity with field, unlike MR fluids, because the magnetic forces in ferrofluids are orders of magnitude smaller.

For chemists everywhere these sort of studies reaffirms that there are areas of CP space still left to be explored, and many fascinating compounds yet to be made and many unusual properties to be discovered. While the past decade has seen much progress in understanding new materials and in using them in commercial products, the field is still laden with opportunities and challenges for interested people working in the CP area.

## Quantum Chemistry of Complex Systems

In 1926, Erwin Schrödinger first derived the analytical solutions for the electronic states of the hydrogen atom. Not long after this, Paul Dirac said: "The underlying physical laws for the mathematical treatment of a large part of physics and the whole chemistry are thus completely known, and the difficulty is only that the exact application of these laws leads to equations much too complicated to be soluble. It therefore becomes desirable that approximate practical methods of applying quantum mechanics should be developed, which can lead to an explanation of the main features of complex atomic systems without too much computation" [11].

What progress has been achieved, some 80 years later, in applying quantum mechanics to complex atomic and molecular systems? Progress in the application of quantum chemistry has depended on advances in computer power. It is now possible, using readily available computer packages, to calculate the energies and properties of many small molecules to an accuracy that can rival that obtained experimentally. This advance has been achieved through the development of theories and computational techniques that provide a rigorous description of electron distribution and electronic correlations. The challenge is to extend these methods and computer programs to solving the Schrödinger equation for systems such as the very large molecules and nanostructures of interest in biology and materials science. The main bottleneck that has prevented accurate electronic structure calculations from being applied to large molecules is the prohibitive scaling of computational cost with the number of electrons, say N, in a molecule. This scaling is N^7 ^for conventional methods, which allow for the correlation of all pairs of electrons in a molecule. However, recently very good news have come in this arena. In fact, some groups have developed powerful methods that allow only electrons close to each other to be correlated and, on its turn, this leads to a linear scaling, that is, a dependence in computational cost on N [12].

The accurate description of electron orbitals in molecules also presents difficulties with the computer time depending on the number of basis functions used to describe an electron (N_A_), because such dependency is of the N_A_^4 ^type. However, another good news have arisen since a recently developed density fitting procedure has reduced this dependence to N_A _[13]. Another advance that improves the accuracy of the calculations is the explicit inclusion of the interelectronic distance in the wave functions used in these approaches [14].

These three fundamental computational developments allow accurate quantum chemistry to be extended to molecular systems with many more atoms than was possible previously. Now it only remains to foresee a large number of contributions applying these and other closely related approaches to face valuable publications on this theoretical chemistry field and we hope that many of them could be registered in *Chemistry Central Journal*.

## Conclusion

Today we are witnessing a rapid and quite impressive rise in the number of new CP contributions with a remarkable diversity of cross-collaborations among quite different chemistry areas. As such, it is my belief that this particular section of the *Chemistry Central Journal *will receive a high number of contributions, and open access will play a significant role in the dissemination of CP information. With this in mind, it will require us to assess properly the real content and significance of each submission received. Having said that, the number of available reviewers in this field is growing, and they are becoming increasingly qualified and as part of this community I hope that you will, when called upon, play your part and do so in a timely manner. It will be necessary to have the author's collaboration to perform a satisfactory editorial work. In this sense, any suggestion or even appropriate criticisms coming from the author or reviewer communities will be acknowledged. It is only working together as reviewers, authors, publishers, and editorial staff that it will be possible to reach the degrees of excellence that the launch of *Chemistry Central Journal *deserves.
